# Evaluation of a Deep Neural Network for Automated Classification of Colorectal Polyps on Histopathologic Slides

**DOI:** 10.1001/jamanetworkopen.2020.3398

**Published:** 2020-04-23

**Authors:** Jason W. Wei, Arief A. Suriawinata, Louis J. Vaickus, Bing Ren, Xiaoying Liu, Mikhail Lisovsky, Naofumi Tomita, Behnaz Abdollahi, Adam S. Kim, Dale C. Snover, John A. Baron, Elizabeth L. Barry, Saeed Hassanpour

**Affiliations:** 1 Department of Biomedical Data Science, Dartmouth College, Hanover, New Hampshire; 2 Department of Computer Science, Dartmouth College, Hanover, New Hampshire; 3 Department of Pathology and Laboratory Medicine, Dartmouth-Hitchcock Medical Center, Lebanon, New Hampshire; 4 Minnesota Gastroenterology PA, Minneapolis; 5 Department of Pathology, Fairview Southdale Hospital, Edina, Minnesota; 6 Department of Medicine, University of North Carolina at Chapel Hill, Chapel Hill; 7 Department of Epidemiology, Dartmouth College, Hanover, New Hampshire

## Abstract

**Importance:**

Histologic classification of colorectal polyps plays a critical role in screening for colorectal cancer and care of affected patients. An accurate and automated algorithm for the classification of colorectal polyps on digitized histopathologic slides could benefit practitioners and patients.

**Objective:**

To evaluate the performance and generalizability of a deep neural network for colorectal polyp classification on histopathologic slide images using a multi-institutional data set.

**Design, Setting, and Participants:**

This prognostic study used histopathologic slides collected from January 1, 2016, to June 31, 2016, from Dartmouth-Hitchcock Medical Center, Lebanon, New Hampshire, with 326 slides used for training, 157 slides for an internal data set, and 25 for a validation set. For the external data set, 238 slides for 179 distinct patients were obtained from 24 institutions across 13 US states. Data analysis was performed from April 9 to November 23, 2019.

**Main Outcomes and Measures:**

Accuracy, sensitivity, and specificity of the model to classify 4 major colorectal polyp types: tubular adenoma, tubulovillous or villous adenoma, hyperplastic polyp, and sessile serrated adenoma. Performance was compared with that of local pathologists’ at the point of care identified from corresponding pathology laboratories.

**Results:**

For the internal evaluation on the 157 slides with ground truth labels from 5 pathologists, the deep neural network had a mean accuracy of 93.5% (95% CI, 89.6%-97.4%) compared with local pathologists’ accuracy of 91.4% (95% CI, 87.0%-95.8%). On the external test set of 238 slides with ground truth labels from 5 pathologists, the deep neural network achieved an accuracy of 87.0% (95% CI, 82.7%-91.3%), which was comparable with local pathologists’ accuracy of 86.6% (95% CI, 82.3%-90.9%).

**Conclusions and Relevance:**

The findings suggest that this model may assist pathologists by improving the diagnostic efficiency, reproducibility, and accuracy of colorectal cancer screenings.

## Key Points

**Question:**

Are deep neural networks trained on data from a single institution for classification of colorectal polyps on digitized histopathologic slides generalizable across multiple external institutions?

**Findings:**

In this prognostic study of a deep neural network to classify the 4 most common polyp types on digitized histopathologic slides from a single institution (internal test set) and 24 US institutions (external test set), the mean accuracy was 93.5% on the internal test set and 87.0% on the external test set.

**Meaning:**

Deep neural networks may provide a generalizable approach for the classification of colorectal polyps on digitized histopathologic slides.

## Teaser

This prognostic study evaluates the performance and generalizability of a deep neural network trained on data from a single institution for classification of colorectal polyps on histopathologic slide images.

## Introduction

In the US, colorectal cancer was estimated to cause 51 020 deaths in 2019, making it the second most common cause of death due to cancer.^1^ This death rate, however, has decreased in the past several decades, likely because of successful cancer screening programs.^2,3,4,5^ Colonoscopy is the most common test in these screening programs in the US.^6^ During colonoscopies, practitioners excise colorectal polyps and visually examine them on histopathologic slides for neoplasia. Early detection of cancer at an early, curable stage and removal of preinvasive adenomas or serrated lesions during this procedure are associated with a reduced mortality rate.^7,8,9^ Furthermore, the numbers and types of polyps detected are associated with the risk of malignant tumors and are therefore used as the basis for subsequent screening recommendations.^6^ An algorithm for automated classification of colorectal polyps could potentially benefit cancer screening programs by improving efficiency, reproducibility, and accuracy as well as reducing the access barrier to pathological services.^10^

In recent years, a class of computational models known as deep neural networks has driven substantial advances in the field of artificial intelligence. Comprising many processing layers, deep neural networks take a data-driven approach to automatically learn the most relevant features of input data for a given task, markedly improving the state of the art in computer vision,^11^ natural language processing,^12^ and speech recognition.^13^ For medical image analysis in particular, deep learning has achieved considerable performance in classification of images, including chest radiographs,^14^ retinal fundus photographs,^15^ head computed tomography scans,^16^ lung histopathologic slides,^17^ and skin cancer images.^18^

This study evaluated the performance and generalizability of a deep neural network for colorectal polyp classification on histopathologic slide images using a multi-institutional data set. To our knowledge, this study is the first to comprehensively evaluate a deep learning algorithm for colorectal polyp classification and assess the generalizability of this model across multiple institutions.

## Methods

### Data Collection

This prognostic study used histopathologic slides from Dartmouth-Hitchcock Medical Center (DHMC), a tertiary academic care center in Lebanon, New Hampshire, to train a deep neural network for colorectal polyp classification. Internal and external data sets of hematoxylin and eosin–stained, formalin-fixed, paraffin-embedded colorectal polyp, whole-slide images were collected. Each of these slides could contain 1 or more tissue section or polyp. This study and the use of human participant data in this project were approved by the Dartmouth-Hitchcock Health Institutional Review Board with a waiver of informed consent. The conducted research reported in this article is in accordance with this approved Dartmouth-Hitchcock Health Institutional Review Board protocol and the World Medical Association Declaration of Helsinki on Ethical Principles for Medical Research Involving Human Subjects.^19^ In addition, the study followed the Standards for Reporting of Diagnostic Accuracy (STARD) reporting guideline.^20^

**Figure 1.**
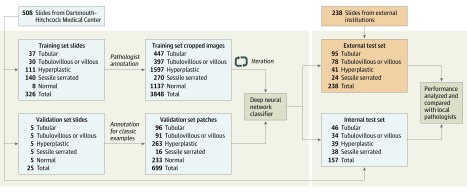
Data Flow Diagram for the Study We trained the model on an internal training and validation set and then evaluated it on internal and external test sets with multipathologist ground truth diagnoses. Annotated regions of interest in the training set varied in length and width, whereas patches in the validation set were of fixed size and represented classic examples of each polyp type.

The internal data set was collected from January 1, 2016, to June 31, 2016, at DHMC. This data set included 508 slides from the 4 most common polyp types according to local diagnoses parsed from pathology reports: tubular adenoma, tubulovillous or villous adenoma, hyperplastic polyp, and sessile serrated adenoma. The slides were scanned (Aperio AT2, Leica Biosystems) at 40× resolution (0.25-μm pixel^−1^) at DHMC. In this internal data set, each whole-slide image was from a different patient and colonoscopy procedure. We partitioned these slides into a training set of 326 slides, a validation set of 25 slides, and an internal test set of 157 slides. The distribution of polyp types was balanced in the validation and internal test sets, whereas slides were oversampled for hyperplastic polyps and sessile serrated adenomas in the training set to improve model training for these classes (Figure 1).

For the external data set, we collaborated with investigators from a randomized clinical trial on the effect of supplementation with calcium and/or vitamin D for the prevention of colorectal adenomas^21^ as well as their network of laboratories. Through this collaboration, we were given access to 1182 whole-slide images along with their diagnoses given by local pathologists. These slides were borrowed from various US pathology laboratories (eTable 1 in the Supplement) by one of us (E.L.B.) from January 1, 2016, to December 31, 2017, and digitized by scanners (Aperio AT2, Leica Biosystems) at 40× resolution at DHMC (similar to the internal data set) before they were returned to the original laboratories. We randomly sampled up to 95 of these slides for each of 4 polyp types as diagnosed by the local pathologist. Of note, 15 of these randomly selected slides were removed because of poor slide quality as determined by our study’s lead expert pathologist (A.A.S.). In total, the final external validation set comprised 238 slides from 24 different institutions in 13 US states. In this external test set, some of the slides corresponded to the same patients because the 238 slides came from 179 distinct patients. All slides from the internal and external test sets were excluded from model development until final evaluation of the model. Each slide in the data set was the most diagnostic slide for the corresponding patient, and slides from the same patient were not from the same lesion.

We did not include any slides with the diagnosis of high-grade dysplasia or adenocarcinoma because we did not have enough samples from these cases in the external validation set. We also did not include normal as a class for whole slides in our study because normal slides are not routinely scanned in the internal or multi-institutional data sets. Moreover, we also did not distinguish regeneration epithelial hyperplasia and inflammatory polyps from hyperplastic polyps and tubular adenomas because of the small number of these cases in our training set. All diagnoses made by DHMC pathologists were based on World Health Organization criteria as of April 2019.^22^

### Data Annotation

The annotation process involved 5 gastrointestinal pathologists (A.A.S., L.J.V., B.R., X.L., M.L.) from the Department of Pathology and Laboratory Medicine at DHMC: 3 (A.A.S., B.R., M.L.) with gastrointestinal pathology fellowship training and 2 (L.J.V., X.L.) who gained gastrointestinal pathology expertise through years of gastrointestinal pathology service. For 157 whole-slide images in the training set, 2 of the gastrointestinal pathologists (A.A.S. and L.J.V.) identified the polyps on the slides and used the Rectlabel^23^ annotation tool to manually annotate rectangular bounding boxes around polyps and normal tissue as regions of interest for model training. In total, 3848 regions of interest were identified and labeled as 1 of the 4 polyp classes.

We also collected a smaller number of annotations from 25 separate whole-slide images as the validation set for hypermetric tuning of the model. In this validation set, the same 2 pathologists (A.A.S. and L.J.V.) annotated nonoverlapping patches of 224 × 224 pixels (or 448 × 448 μm) of classic examples for each polyp type. Because this data set was used to guide model development, all fixed-size patches were confirmed with high confidence by both pathologists, and patches with disagreements were discarded.

For the internal test set, the 5 gastrointestinal pathologists independently and retrospectively made a diagnosis based on each slide as 1 of the 4 polyp types. For this internal set, the local diagnoses given at DHMC may have been from 1 of the 5 study gastrointestinal pathologists, but the original diagnosis and identity of the pathologist at the point of care were hidden during the retrospective annotation phase.

For the external test set, the 5 gastrointestinal pathologists from DHMC also retrospectively made diagnoses based on all slides in the test set in the same fashion as for the internal test set. In total, 5 complete sets of diagnoses from gastrointestinal pathologists and the diagnoses given by local pathologists at the point of care were recorded. For both the internal and the external test sets, ground truth diagnoses were assigned by taking the majority vote of the 5 gastrointestinal pathologists. Figure 1 depicts the data flow for the study design. eFigure 1 in the Supplement shows the statistics on polyp types, number of patches, and slide sizes for the internal and external test sets.

### Deep Learning Model

In this study, we implemented the deep residual network (ResNet), a neural network architecture that significantly outperformed all other models on the ImageNet and Common Objects in Context image recognition benchmarks.^24^ For model training, we applied a sliding window method to the 3848 variable-size regions of interest labeled by pathologists in the training set, extracting approximately 7000 fixed-size 224 × 224-pixel patches per polyp type. Then, we initialized ResNet with the MSRA (Microsoft Research Asia) weight initialization^11^ and trained the neural network for 200 epochs with an initial learning rate of 0.001, which decayed by a factor of 0.9 every epoch. Throughout training, we applied standard image augmentation techniques, including rotations and flips as well as color jittering on the brightness, contrast, saturation, and hue of each image. For our final model, we used an ensembled model that comprised 5 ResNets of 18, 34, 50, 101, and 152 layers. Overall, training these networks took approximately 96 hours using a single graphics processing unit (NVIDIA Tesla K40c). Once the model was trained, there was no further modification of the model based on the pathologists’ examination of the results.

### Slide-Level Inference

For the deep learning model to infer the overall diagnosis of a whole-slide image, we designed a hierarchical classification algorithm to match the nature of the classification task. Each slide was initially broken down into many patches using a sliding window algorithm, and each patch was classified by the neural network.

Using the predicted diagnoses by the neural network for all patches in a given slide, the model first determined whether a polyp was adenomatous (tubular, tubulovillous, or villous) or serrated (hyperplastic or sessile serrated) by comparing the number of predicted patches for the adenomatous and serrated types. Adenomatous polyps with more than a certain amount of tubulovillous or villous tissue (>30%) were classified as overall tubulovillous or villous adenoma, whereas the remaining polyps were classified as tubular adenoma. For serrated polyps, the algorithm classified polyps with above a certain amount of sessile serrated patches (>1.5%) as overall sessile serrated adenomas and the remaining polyps as hyperplastic. All thresholds were determined using a grid search over the internal training set. The hierarchical nature of the inference heuristic allowed us to imitate the schema used by pathologists for this classification task without training a separate machine learning classifier.

### Evaluation

For final evaluation, we compared the performance of the model with that of local pathologists originally made at the point of care on the internal test set and the multi-institutional external test set. Local pathologist performance measures were averaged over all samples because information about individual pathologists’ performances were anonymized. To assess the quality of annotations in our study, we measured the agreement of our gastrointestinal pathologists in terms of multiclass Cohen κ. The application of the final model on a whole-slide image in the test sets took less than a mean of 60 seconds using a single graphics processing unit (NVIDIA Tesla K40c). For the model’s classifications, we calculated accuracy, sensitivity, and specificity in comparison with ground truth diagnoses and compared these metrics with those of local pathologists. Furthermore, we calculated confusion matrixes for local pathologists and the model and conducted appropriate error analysis.

### Statistical Analysis

The algorithms in this study were implemented in Python software, version 3.6 (Python Software Foundation). We used OpenSlide software, version 3.4.1 (Carnegie Mellon University School of Computer Science) to convert the digitized image format and PyTorch software, version 0.4 (Facebook’s AI Research Lab) for training the deep neural network models. The statistical analysis and 95% CIs were calculated using the Statistics, version 3.4 library in Python. The source code for this study is publicly available.^25^

We used a 2-tailed *t* test for proportions with a significance level of 2-sided *P* ≤ .05 to compare the performance of local pathologists and the model on the internal and external test sets. R, version 3.3.3 (R Foundation for Statistical Computing) was used for the statistical analysis in this study. Data analysis was performed from April 9 to November 23, 2019.

## Results

### Internal Evaluation

The Table gives the per-class and mean performance metrics of local pathologists and the proposed model for internal and external test sets. For the internal test set from DHMC, interobserver agreement, measured by Cohen κ, was in the substantial range of 0.61 to 0.80, with the 5 study gastrointestinal pathologists achieving a mean multiclass Cohen κ of 0.72 (95% CI, 0.64-0.80). The model achieved a mean accuracy (the unweighted mean of individual polyp type accuracies) of 93.5% (95% CI, 89.6%-97.4%) compared with local pathologists’ accuracy of 91.4% (95% CI, 87.1%-95.8%) on the internal data set. A 2-tailed *t* test for proportions revealed, however, that the differences in performance were not significant (pathologist, 91.4%; deep neural network, 93.5%; *P* = 0.50 for accuracy; pathologist, 80.7%; deep neural network, 86.8%; *P* = .14 for sensitivity; and pathologist, 95.1%; deep neural network, 95.7%; *P* = .80 for specificity).

**Table.** Per-Class Comparison Between Local Pathologists and the Deep Neural Network Model in Classifying Colorectal Polyps on Internal and External Test Sets

Polyp type	Internal test set (n = 157)						External test set (n = 238)					
	Local pathologists			Deep neural network			Local pathologists			Deep neural network		
	Accuracy, %	Sensitivity, %	Specificity, %	Accuracy, %	Sensitivity, %	Specificity, %	Accuracy, %	Sensitivity, %	Specificity, %	Accuracy, %	Sensitivity, %	Specificity, %
TA	89.8	76.1	95.5	93.0	89.1	94.6	79.8	53.7	97.2	84.5	73.7	91.6
TVA	94.3	88.2	95.8	95.5	97.1	95.1	81.5	100	77.7	89.5	97.6	87.8
HP	89.8	76.9	94.1	92.4	82.1	95.8	91.6	80.8	96.8	85.3	60.3	97.5
SSA	91.7	81.6	95.0	93.0	78.9	97.5	93.3	79.2	94.8	88.7	79.2	89.7
Mean	91.4	80.7	95.1	93.5	86.8	95.7	86.6	78.4	91.6	87.0	77.7	91.6

Abbreviations: HP, hyperplastic polyp; SSA, sessile serrated adenoma; TA, tubular adenoma; TVA, tubulovillous or villous adenoma.

### Multi-institutional External Evaluation

The external data set had less agreement for pathologists and the model. The 5 study gastrointestinal pathologists achieved a mean multiclass Cohen κ of 0.67 (95% CI, 0.60-0.75). With an accuracy of 87.0% (95% CI, 82.7%-91.3%) on the external test set, the model performed at a similar level of accuracy, sensitivity, and specificity as local pathologists on this data set (pathologist, 86.6%; deep neural network, 87.0%; *P* = .90 for accuracy; pathologist, 78.4%; deep neural network, 77.7%; *P* = .86 for sensitivity; and pathologist, 91.6%; deep neural network, 91.6%; *P* = .99 for specificity). The Table gives the performance metrics for local pathologists and deep neural network for each polyp class on the internal and external test sets. eTable 2 in the Supplement gives the performance of local pathologists and the deep learning model stratified by the agreement of DHMC pathologists in determining ground truth labels.

### Confusion Matrices and Error Analysis

**Figure 2.**
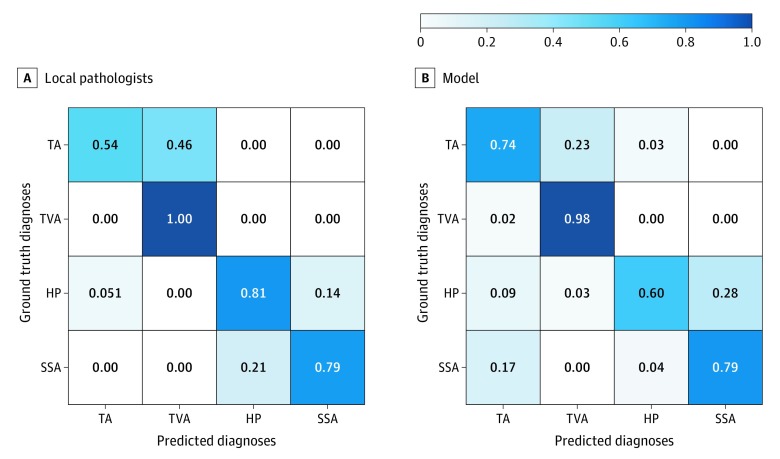
Confusion Matrixes for Local Pathologists’ Diagnoses Given at the Point of Care and the Model’s Predicted Diagnoses in Comparison With Multipathologist Ground Truth Diagnoses for the External Test Set Each cell in the confusion matrix is the agreement ratio between multipathologist ground truth labels and local pathologists’ or the model’s diagnoses. HP indicates hyperplastic polyp; SSA, sessile serrated adenoma; TA, tubular adenoma; and TVA, tubulovillous or villous adenoma.

Moreover, in Figure 2, we calculated confusion matrixes for local pathologists and the model on the external test set to determine which polyp types were the most challenging to diagnose. Local pathologists often classified tubular adenomas as tubulovillous or villous adenomas (46.3%) and hyperplastic polyps as sessile serrated adenomas (12.9%). The deep neural network similarly classified many tubular adenomas as tubulovillous or villous adenomas (23.2%) and hyperplastic polyps as sessile serrated adenomas (27.3%). For further analysis of the model’s errors, eFigure 2 in the Supplement shows violin plots for predicted percentage areas of each polyp type on slides.

### Visualization

**Figure 3.**
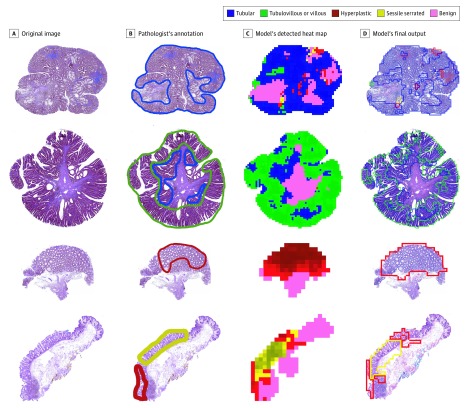
Visualization of the Classifications of the Deep Neural Network Model In the model’s detected heat map, the higher confidence predictions are shown in darker color. The model’s final output highlights precancerous lesions that can potentially be used to aid pathologists in clinical practice.

The results of the model were visualized on digitized slides by highlighting the regions that contributed to the whole-slide classification. Figure 3 shows examples of slides with the lead gastrointestinal pathologist’s (A.A.S.) annotations, the heat map detected by the model, and the visualization of our model’s results.

## Discussion

To our knowledge, this study is the first to evaluate a deep neural network for colorectal polyp classification on a large multi-institutional data set with comparison with local diagnoses made at the point of care. On a test set of 238 images from 24 external institutions, the model achieved an accuracy of 87.0%, which was on par with the local pathologists’ accuracy of 86.6% at the α = .05 level. With regard to annotation agreement, the 5 study gastrointestinal pathologist annotators had a mean Cohen κ of 0.72 on the internal test set and 0.67 on the external test set, which were higher than the previously reported Cohen κ scores of 0.46,^26^ 0.31,^27^ 0.55,^28^ and 0.54.^29^ This difference in performance is likely attributable to differences in polyp type distributions in various data sets, interlaboratory variations in tissue processing and staining, and institutional biases in the polyp classification criteria. Of note, although including the external slides for training would likely improve the performance of the model on the external test set, the deep neural network was intentionally trained only on the internal data set to examine its generalizability to external institutions.

In terms of error analysis, the deep neural network made similar misclassifications as local pathologists, as shown by the similarities in their confusion matrixes. Both the model and the local pathologists distinguished adenomatous (tubular, tubulovillous, or villous) and serrated (hyperplastic or sessile serrated) polyps with high accuracy, whereas the model had a higher number of mistakes within those 2 categories. Of note, the model used a simple hierarchical heuristic based on the number of predicted patches to distinguish adenomatous and serrated polyps on a whole slide, which is not as nuanced as a pathologist’s line of thought in real-world settings. Further subclassification of adenomatous and serrated polyps was relatively more challenging for the model. We hypothesize that many of the mistakes occurred because thresholds for detection of tubulovillous or villous growths and of sessile serrated crypts vary among pathologists because the lead gastrointestinal pathologist’s manual inspection of discordances found that many of the errors made by the deep neural network were similar to mistakes made by pathologists in practice. For example, a common mistake made by both the model and the local pathologists was distinguishing hyperplastic polyps and sessile serrated adenomas, potentially reflecting the data imbalance of the sessile serated adenoma class in the training set.

This study not only showed the utility of a deep learning model for classification of colorectal polyps but also advances previous literature^14,15,16,17,18,30,31,32,33^ in terms of model evaluation and study design. A previous study on deep learning for colorectal polyp classification^30,31^ demonstrated good performance on an internal data set but used a simpler approach and did not include pathologist-level performance or local diagnoses. The present study, on the other hand, evaluated a deep neural network on a multi-institutional external data set and demonstrated a comparable diagnostic performance of deep neural networks compared with local pathologists at the point of care. Many previous studies^14,15,16,17,18,32,33^ demonstrated practitioner-level performance of deep neural networks on various medical classification tasks. All these studies,^14,15,16,17,18,30,31,32,33^ however, measured practitioner-level performance on a predetermined number of practitioners from a few medical institutions in a controlled setting. Although it is important to measure retrospective practitioner performance on classification tasks, we used diagnoses by local pathologists in clinical practice at the point of care in 24 external institutions for comparison against the deep neural network.

A deep learning model for colorectal polyp classification, if validated through clinical trials, has potential for widespread application in clinical settings. Our model could be implemented in laboratory information systems to guide pathologists by identifying areas of interest on digitized slides, which could improve work efficiency, reproducibility, and accuracy for colorectal polyp classification. Although expert practitioner confirmation of diagnoses will still be required, the model could help triage slides indicating diagnoses that are more likely to be preinvasive for subsequent review by pathologists. Because the US Preventive Services Task Force recommends that all adults aged 50 to 75 years undergo screening for colorectal cancer, an automated model for classification could be useful in relieving pathologists’ burden in slide review and ultimately reduce the barrier of access for colorectal cancer screening.

Moving forward, further work can be performed in deep learning for analysis of colorectal polyp images. Foremost, we plan to implement the model prospectively in a clinical setting to measure its ability to enhance pathologists’ classification of colorectal polyps and improve outcomes in a clinical trial. In terms of technical improvements to the model, more data can be collected and used for training to increase the model’s performance, especially for sessile serrated adenomas, and new less common classes, such as high-grade dysplasia, adenocarcinoma, regeneration epithelial hyperplasia, and inflammatory polyps. Moreover, related work has found that deep learning can identify hidden features in histopathologic images that can be used to detect gene mutations^17^ and predict patient survival,^34,35,36^ tasks that pathologists do not perform. To this end, we plan to collect more patient outcome data to train the model to predict polyp recurrence and patient survival in colorectal cancer.

### Limitations

This study has limitations. Although the model performed on par with local pathologists on the external test set, it did not perform as well as the internal evaluation. The results suggest that there is a higher level of variability among slides from various institutions and the model could be further improved by training on larger, diverse data sets. Furthermore, although the model identified the most common polyp types, the study was performed on well-sectioned, clearly stained slides and did not include less common classes, such as traditional serrated adenoma or sessile serrated adenoma with cytologic dysplasia. In addition, the model was not evaluated on entirely normal slides. Our team plans to collect further data and extend the model and its evaluation to these additional cases as future work. In addition, local pathologists might have had access to additional slides and patient information, such as patient colonoscopy history and polyp biopsy location, that may have influenced their diagnoses. Access to this additional information might explain some of the discrepancies between local diagnoses and ground truth labels, which were only based on digitized slides.

## Conclusions

 In this study, the performance of the deep learning model was similar to that of local pathologists on the internal and external test sets. If confirmed in clinical trials, this model could improve the efficiency, reproducibility, and accuracy of colonoscopy.

## Notes

Supplement. **eTable 1.** Colorectal Polyp Slide Class Distribution for our Multi-Institutional External Test Set Grouped by Pathology Laboratory Institutional Affiliation Type and State **eTable 2.** Performance of Local Pathologists and Our Deep Neural Network Stratified by Level of Agreement of the Five DHMC Pathologists for Ground-Truth Labels for the Multi-Institutional External Validation Set of 238 Slides **eFigure 1.** Number of Patches per Digitized Slide and Slide Size (in Pixels) for (A) the Internal Test Set and (B) the Multi-institutional External Test Set **eFigure 2.** Violin Plots Showing Predicted Percentage Areas (Based on Number of Patches) for Each Polyp Type on Whole-Slide Images, Depicting the Distribution of Predicted Patches by the Model for Corresponding Ground Truth Labels
