# Supplementary material for: Evaluation of a Deep Neural Network for Automated Classification of Colorectal Polyps on Histopathologic Slides
Source: JAMA Netw Open. 2020 Apr 23;3(4):e203398. doi: 10.1001/jamanetworkopen.2020.3398 (PMC7180424; doi:10.1001/jamanetworkopen.2020.3398)
Supplement: Supplement. — eTable 1. Colorectal Polyp Slide Class Distribution for our Multi-Institutional External Test Set Grouped by Pathology Laboratory Institutional Affiliation Type and State eTable 2. Performance of Local Pathologists and Our Deep Neural Network Stratified by Level of Agreement of the Five DHMC Pathologists for Ground-Truth Labels for the Multi-Institutional External Validation Set of 238 Slides eFigure 1. Number of Patches per Digitized Slide and Slide Size (in Pixels) for (A) the Internal Test Set and (B) the Multi-institutional External Test Set eFigure 2. Violin Plots Showing Predicted Percentage Areas (Based on Number of Patches) for Each Polyp Type on Whole-Slide Images, Depicting the Distribution of Predicted Patches by the Model for Corresponding Ground Truth Labels [file jamanetwopen-3-e203398-s001.pdf]

Supplementary Online Content

Wei JW, Suriawinata AA, Vaickus LJ, et al. Evaluation of a deep neural network for automated classification of colorectal polyps on histopathologic slides. *JAMA Netw Open*. 2020;3(4):e203398. doi:10.1001/jamanetworkopen.2020.3398

eTable 1. Colorectal Polyp Slide Class Distribution for our Multi-Institutional External Test Set Grouped by Pathology Laboratory Institutional Affiliation Type and State

eTable 2. Performance of Local Pathologists and Our Deep Neural Network Stratified by Level of Agreement of the Five DHMC Pathologists for Ground-Truth Labels for the Multi-Institutional External Validation Set of 238 Slides

eFigure 1. Number of Patches per Digitized Slide and Slide Size (in Pixels) for **(A)** the Internal Test Set and **(B)** the Multi-institutional External Test Set

eFigure 2. Violin Plots Showing Predicted Percentage Areas (Based on Number of Patches) for Each Polyp Type on Whole-Slide Images, Depicting the Distribution of Predicted Patches by the Model for Corresponding Ground Truth Labels

This supplementary material has been provided by the authors to give readers additional information about their work.

© 2020 Wei JW et al. *JAMA Network Open*.

eTable 1. Colorectal Polyp Slide Class Distribution for our Multi-Institutional External Test Set Grouped by Pathology Laboratory Institutional Affiliation Type and State

Pathology Lab Affiliation Type	State	Pathologist Consensus Diagnosis				
		TA	TVA	HP	SSA	Total Slides
University hospital	Georgia	3	0	25	2	30
	Colorado	0	4	7	0	11
	California	3	0	0	0	3
	Texas	0	0	1	0	1
Veteran's hospital	Georgia	17	1	0	0	18
	Minnesota	6	3	1	0	10
	Colorado	1	0	4	0	5
Metropolitan/regional hospital	Iowa	6	3	3	2	14
	Ohio	4	1	6	5	16
	Minnesota	0	0	2	5	7
	South Carolina	3	1	3	1	8
	New Hampshire	4	0	1	0	5
	South Carolina	2	0	0	0	2
	Minnesota	0	0	0	1	1
	California	2	0	0	0	2
	Iowa	1	0	1	0	2
Freestanding	Iowa	15	3	4	1	23
	Iowa	10	9	5	0	24
	Iowa	2	0	3	0	5
	Colorado	0	0	1	0	1
	New Hampshire	1	0	2	0	3
	Colorado	0	0	3	0	3
	Florida	0	0	1	0	1
Specialty clinic/practice	Minnesota	15	16	5	7	43
Combined		95	41	78	24	238

TA: tubular adenoma; TVA: tubulovillous/villous adenoma; HP: hyperplastic polyp; SSA: sessile serrated adenoma.

© 2020 Wei JW et al. *JAMA Network Open*.

eTable 2. Performance of Local Pathologists and Our Deep Neural Network Stratified by Level of Agreement of the Five DHMC Pathologists for Ground-Truth Labels for the Multi-Institutional External Validation Set of 238 Slides

	Number of Slides					Local Pathologists			Deep Neural Network		
Level of Agreement	TA	TVA	HP	SS	Total	Acc	Sens (%)	Spec	Acc	Sens (%)	Spec
3/5 Pathologists	19	14	9	4	46	79.3	64.2	86.0	80.4	65.6	87.0
4/5 Pathologists	19	10	19	7	55	83.6	71.0	89.4	82.7	72.7	89.2
5/5 Pathologists	57	17	49	14	137	90.5	85.5	94.3	91.2	83.2	94.1

Both local pathologists and the deep neural network performed better on slides that more DHMC pathologists agreed on. Acc: unweighted average class accuracy; Sens: unweighted average class sensitivity; Spec: unweighted average class specificity. TA: tubular adenoma; TVA: tubulovillous/villous adenoma; HP: hyperplastic polyp; SSA: sessile serrated adenoma.

© 2020 Wei JW et al. *JAMA Network Open*.

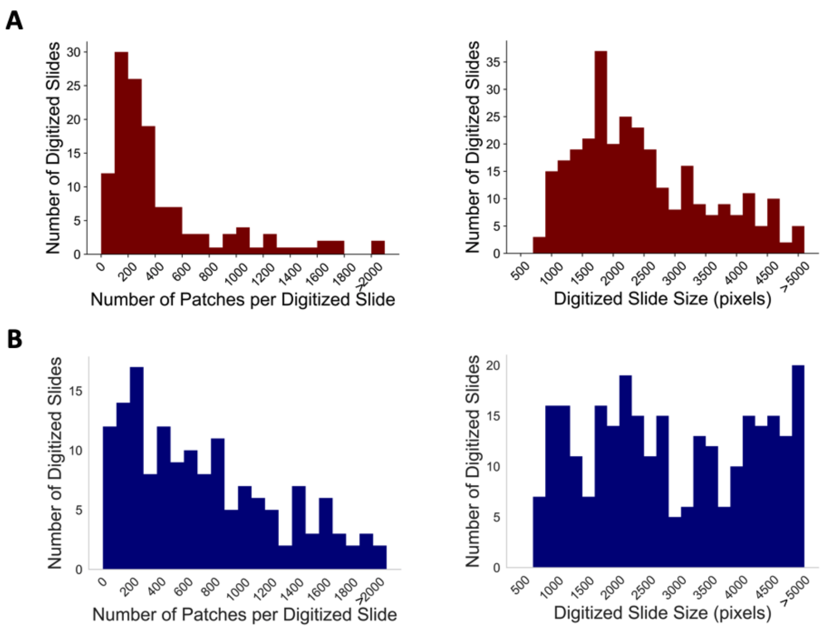

eFigure 1. Number of Patches per Digitized Slide and Slide Size (in Pixels) for **(A)** the Internal Test Set and **(B)** the Multi-institutional External Test Set

Patches are fixed-size areas of tissue obtained by sliding a window over the entire image. Size of digitized slides reflects the area of the tissue after removing the background.

© 2020 Wei JW et al. *JAMA Network Open*.

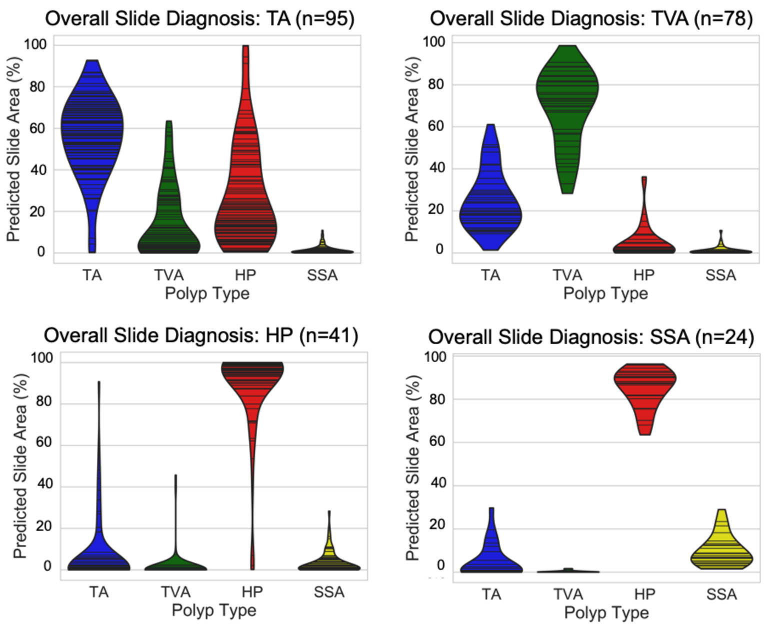

eFigure 2. Violin Plots Showing Predicted Percentage Areas (Based on Number of Patches) for Each Polyp Type on Whole-Slide Images, Depicting the Distribution of Predicted Patches by the Model for Corresponding Ground Truth Labels

TA: tubular adenoma; TVA: tubulovillous/villous adenoma; HP: hyperplastic polyp; SSA: sessile serrated adenoma. For whole slides that were diagnosed as TA and TVA, our model detected significant areas of TA and TVA, reflecting the morphological similarity of the two polyp types. Our model also detected large areas of HP in whole slides diagnosed as TA, which is expected since all polypoid lesions in TAs are exposed to elevated mechanical forces and therefore show hyperplastic features at their peripheries. While the model detected mostly HP areas in whole slides diagnosed as hyperplastic polyps, it did find some small areas of SSAs, possibly because larger HPs with deep, dilated crypts and serrated epithelium can appear similar to SSAs. Finally, for whole slides that were diagnosed as SSAs, it makes sense that HPs comprised the largest area, since all SSAs have significant morphological overlap with

© 2020 Wei JW et al. *JAMA Network Open*.

hyperplastic polyps and may contain only a few classic, broad-based, dilated crypts with heavy serration.

© 2020 Wei JW et al. *JAMA Network Open*.
